# Comparing one dose of HPV vaccine in girls aged 9–14 years in Tanzania (DoRIS) with one dose in young women aged 15–20 years in Kenya (KEN SHE): an immunobridging analysis of randomised controlled trials

**DOI:** 10.1016/S2214-109X(23)00586-7

**Published:** 2024-02-14

**Authors:** Kathy Baisley, Troy J Kemp, Nelly R Mugo, Hilary Whitworth, Maricianah A Onono, Betty Njoroge, Jackton Indangasi, Elizabeth A Bukusi, Priya R Prabhu, Paul Mutani, Denise A Galloway, David Mwanzalime, Saidi Kapiga, Charles J Lacey, Richard J Hayes, John Changalucha, Ligia A Pinto, Ruanne V Barnabas, Deborah Watson-Jones

**Affiliations:** a Faculty of Epidemiology and Population Health, London School of Hygiene & Tropical Medicine, London, UK; b Faculty of Infectious and Tropical Diseases UK, London School of Hygiene & Tropical Medicine, London, UK; c HPV Serology Laboratory, Frederick National Laboratory for Cancer Research, Frederick, MD, USA; d Department of Global Health, University of Washington, Seattle, WA, USA; e Center for Clinical Research, Kenya Medical Research Institute, Nairobi, Kenya; f Mwanza Intervention Trials Unit, National Institute for Medical Research, Mwanza, Tanzania; g Center for Microbiology Research, Kenya Medical Research Institute, Nairobi, Kenya; h Human Biology Division, Fred Hutchinson Cancer Center, Seattle, WA, USA; i York Biomedical Research Institute & Hull York Medical School, University of York, York, UK; j Division of Infectious Diseases, Massachusetts General Hospital, Boston, MA, USA

## Abstract

**Background:**

The first randomised controlled trial of single-dose human papillomavirus (HPV) vaccine efficacy, the Kenya single-dose HPV-vaccine efficacy (KEN SHE) trial, showed greater than 97% efficacy against persistent HPV16 and HPV18 infection at 36 months among women in Kenya. We compared antibody responses after one dose of HPV vaccine in the Dose Reduction Immunobridging and Safety Study (DoRIS), the first randomised trial of the single- dose regimen in girls aged 9–14 years, the target age range for vaccination, with those after one dose of the same vaccine in KEN SHE.

**Methods:**

In the DoRIS trial, 930 girls aged 9–14 years in Tanzania were randomly assigned to one, two, or three doses of the 2-valent vaccine (Cervarix) or the 9-valent vaccine (Gardasil-9). The proportion seroconverting and geometric mean concentrations (GMCs) at month 24 after one dose were compared with those in women aged 15–20 years who were randomly assigned to one dose of the same vaccines at the same timepoint in KEN SHE. Batched samples were tested together by virus-like particle ELISA for HPV16 and HPV18 IgG antibodies. Non-inferiority of GMC ratios (DoRIS trial:KEN SHE) was predefined as a lower bound of the 95% CI less than 0·50.

**Findings:**

Month 24 HPV16 and HPV18 antibody GMCs in DoRIS were similar or higher than those in KEN SHE. 2-valent GMC ratios were 0·90 (95% CI 0·72–1·14) for HPV16 and 1·02 (0·78–1·33) for HPV18. 9-valent GMC ratios were 1·44 (95% CI 1·14–1·82) and 1·47 (1·13–1·90), respectively. Non-inferiority of antibody GMCs and seropositivity was met for HPV16 and HPV18 for both vaccines.

**Interpretation:**

HPV16 and HPV18 immune responses in young girls 24 months after a single dose of 2-valent or 9-valent HPV vaccine were comparable to those in young women who were randomly assigned to a single dose of the same vaccines and in whom efficacy had been shown. A single dose of HPV vaccine, when given to girls in the target age range for vaccination, induces immune responses that could be effective against persistent HPV16 and HPV18 infection at least two years after vaccination.

**Funding:**

The UK Department of Health and Social Care, the Foreign, Commonwealth, & Development Office, the Global Challenges Research Fund, the UK Medical Research Council and Wellcome Trust Joint Global Health Trials scheme, the Bill and Melinda Gates Foundation, the US National Cancer Institute; the US National Institutes of Health, and the Francis and Dorothea Reed Endowed Chair in Infectious Diseases.

**Translation:**

For the KiSwahili translation of the abstract see Supplementary Materials section.

## Introduction

Cervical cancer is the leading cause of cancer-related morbidity and mortality among women in much of sub-Saharan Africa. It is caused by infection with oncogenic human papillomavirus (HPV) genotypes, which is almost entirely preventable through prophylactic vaccination. As part of its global strategy for cervical cancer elimination, WHO has set a target of 90% of girls worldwide being vaccinated against HPV by the age of 15 years by 2030.^1^ However, in 2021, only 21% of girls aged 15 years were estimated to be vaccinated against HPV, largely because many countries had not yet introduced HPV vaccination programmes.^2^ A major barrier to introduction, particularly for low-income and middle-income countries, has been the costs and logistical challenges of delivering the vaccine as a multi-dose schedule.^3^ A global shortage of HPV vaccine also contributed to delays in its introduction for some countries.

Over the past decade, accumulating observational evidence has suggested that a single dose of HPV vaccine produces durable immune responses and protection against HPV infection and cervical cancer precursor lesions. Observational studies nested within three large HPV vaccine trials in which some participants did not complete their allocated dose schedules (ie, the Costa Rica Vaccine trial (CVT), the PATRICIA trial, and the IARC/India trial) have shown comparable efficacy against persistent HPV infection, a necessary precursor for cervical cancer, in females who received one dose compared with those who received two or three doses.^4^, ^5^, ^6^

**Research in context**

**Evidence before this study**

A review of the evidence for single dose human papillomavirus (HPV) vaccination was published in November, 2020, by the Single-Dose HPV Vaccine Evaluation Consortium, which summarised papers published until Aug 10, 2020. Results included eight observational studies that arose from three randomised trials (ie, Costa Rica Vaccine Trial (CVT), PATRICIA, and IARC/India HPV vaccine trials) in which participants received fewer than their allocated doses, showing that single dose antibody concentrations stabilised around 12 months after vaccination at a plateau level that was maintained and provided protection against persistent HPV infection for up to 11 years. Immunogenicity results were also available from 11 observational studies of women who received fewer than three doses through national HPV vaccination programmes, similarly showing that antibody concentrations after one dose were considerably higher than for natural infection and were sustained over time. Lastly, in 21 observational studies of vaccine effectiveness against HPV infection or cervical abnormalities among partially vaccinated women, ten found some evidence of effectiveness of one dose. On July 16, 2023, we searched PubMed for papers published since Aug 10, 2020, using the terms “human papillomavirus” AND “vaccine” AND (“immunogenicity” OR “efficacy” OR “effectiveness”) AND “single dose”. This search identified ten studies, five of which extended results from the CVT and IARC/India/ trials. The others included a study of women in Fiji who were vaccinated through a national HPV vaccination campaign in 2008–09, which showed 81% vaccine effectiveness of one dose against prevalent HPV16 and HPV18 infection. A non-randomised immunogenicity trial in the USA of a delayed second dose of Gardasil-9 among girls and boys aged 9–11 years showed that HPV16 and HPV18 antibody concentrations remained stable up to 24 months after one dose. The remaining three papers reported the results of the KEN SHE trial, the first randomised trial of single dose HPV vaccine efficacy, in females aged 15–20 years in Kenya, and the immunogenicity and immunobridging results of the DoRIS trial in Tanzania, the first randomised trial of the one-dose schedule in girls in the target age range for HPV vaccination (ie, aged 9–14 years). The KEN SHE trial showed one dose vaccine efficacy of 97*·*5%. The DoRIS trial showed that more than 98% of girls who received one dose were seropositive for HPV16 and HPV18 IgG antibodies at 24 months, had antibody concentrations that were stable over time, and who were non-inferior to those who had received one dose in the CVT and India/IARC trials.

**Added value of this study**

Here we present an immunobridging study comparing immune responses in the DoRIS trial with those in the KEN SHE trial. Both trials were conducted in east Africa, a region with one of the highest cervical cancer rates worldwide, and included enrolment of participants from malaria-endemic localities. Since evaluating HPV vaccine efficacy in young girls is difficult because of the time needed to accrue HPV infection endpoints, immunobridging studies are valuable for inferring protection in the young population. The trials used the same two HPV vaccines; this study provides the first immunobridging efficacy results for the 9-valent vaccine. We show that HPV16 and HPV18 antibody concentrations and seropositivity at 24 months after a single dose of HPV vaccine in girls aged 9–14 years in the DoRIS trial were non-inferior to those in young females aged 15–20 years in the KEN SHE trial. These results are salient for low-income settings where the cost and logistical advantages of a single dose regimen are particularly important.

**Implications of all the available evidence**

These results from the first two randomised trials of the single-dose schedule provide the strongest available evidence that one dose of HPV vaccine induces immune responses in young girls that are comparable with those seen in young women in whom efficacy has been shown, and are sustained for up to two years after vaccination. These data add to the observational evidence showing efficacy of a single dose up to 11 years, and further support the recent WHO recommendation for a single dose HPV vaccine schedule, providing a promising strategy towards achieving cervical cancer elimination.

Recently, these observational findings have been confirmed in two of the first randomised controlled trials of one dose of HPV vaccine, both in sub-Saharan Africa. The Kenya single-dose HPV-vaccine efficacy (KEN SHE) trial, conducted among sexually-active women aged 15–20 years, found that a single dose of the 2-valent vaccine (Cervarix, GlaxoSmithKline Biologicals, Rixensart, Belgium) or of the 9-valent vaccine (Sanofi Pasteur MSD, Lyon, France) provided 97·5% or higher efficacy against incident 6-month persistent HPV16 and HPV18 infection, compared with a control vaccine.^7^ The Dose Reduction Immunobridging and Safety Study (DoRIS) trial, conducted among girls aged 9–14 years (the target age group for HPV vaccination) in Tanzania, found that more than 98% of participants were seropositive for antibodies to HPV16 and HPV18 at 24 months after vaccination with either the 2-valent or 9-valent vaccines, irrespective of whether they received one, two, or three doses, and antibody concentrations after a single dose were stable for up to 3 years.^8^, ^9^ HPV16 and HPV18 antibody concentrations after one dose of HPV vaccine in DoRIS were found to be non-inferior to those who had received one dose in the CVT and IARC/India trials, in whom efficacy has been shown for up to 11 years or more.^10^ Given the strength of the available evidence, WHO recently amended their recommendations regarding HPV vaccination to allow either one-dose or two-dose schedules among individuals who are immunocompetent up to 20 years of age.^11^

Here we aimed to report the results of immunobridging the DoRIS to KEN SHE trials, comparing immune responses after one dose in young girls in Tanzania with those in young women in Kenya. These results provide several important advances over previous immunobridging comparisons. First, we are bridging immune responses in DoRIS to a randomised trial with direct and rigorous evidence of efficacy rather than to observational studies, thus providing the strongest available evidence of efficacy of the single- dose regimen in young girls. The KEN SHE and DoRIS trials used the same two vaccines, so provide the first immunobridging comparison for the 9-valent vaccine. The trials were both conducted in east Africa, which has one of the highest rates of cervical cancer in the world, and where the cost and logistical advantages of a single-dose regimen are particularly important. Lastly, the DoRIS trial and one of the KEN SHE trial sites were in malaria-endemic areas; malaria has been shown to affect the immune responses to some vaccines.^12^, ^13^ The first trial of HPV vaccine in Africa showed some evidence of higher antibody concentrations in participants with malaria than in participants without malaria.^14^ In 2014, WHO called for HPV vaccine trials in malaria-endemic areas as a research priority.^15^

## Methods

### Study design

The DoRIS trial (NCT02834637) is the first randomised trial to evaluate the immunogenicity of a single-dose regimen in girls in the target age range for vaccination. The trial commenced enrolment in February, 2017, and has been described in detail previously.^16^ In brief, it is an ongoing unblinded, individually randomised controlled trial comparing reduced-dose schedules of two HPV vaccines among 930 healthy, HIV-negative Tanzanian schoolgirls aged 9–14 years (appendix 2 p 1). Girls were randomly allocated to one of six groups comprising three doses, two doses, or a single dose of the GlaxoSmithKline 2-valent or Merck 9-valent vaccine. All participants were followed up to month 36; girls in the one dose and two dose groups have been enrolled in a trial extension and will be followed up to 9 years.

### Immunobridging objectives

The overall aim of the immunobridging study is to compare vaccine-induced HPV-specific immune responses 24 months after one dose in young girls in the DoRIS trial with those after one dose in young women in the KEN SHE trial. Our hypothesis was that HPV16 and HPV18 antibody responses in girls aged 9–14-years after a single dose of HPV vaccine would be non-inferior to those observed in young women aged 15–20-years.

The primary objective was to show non-inferiority of HPV16 and HPV18 antibody geometric mean concentrations (GMCs) at month 24 after vaccination, when comparing one dose of HPV vaccine in the DoRIS trial with one dose of the same vaccine in KEN SHE. The secondary objective was to show non-inferiority of HPV16 and HPV18 seropositivity. The month 24 timepoint was chosen because studies have shown that immune responses after a single dose in females aged 10–25 years reach a plateau around 12 months, after which they remain stable up to 11 years.^6^, ^17^ Results from the DoRIS trial showed that antibody concentrations after one dose declined between month 1 and month 7 then reached a plateau by month 12 and remained stable up to month 36.^8^

### The KEN SHE trial

KEN SHE is the first randomised controlled efficacy trial of a single dose of HPV vaccine. The trial enrolled 2275 healthy, HIV-negative, sexually active young women aged 15–20 years in Kenya between December, 2018, and November, 2019.^18^ Women were randomly allocated to one of three groups, comprising a single dose of the GlaxoSmithKline 2-valent HPV vaccine, the Merck 9-valent HPV vaccine, or meningococcal vaccine (appendix 2 p 1). Women enrolled in the main trial were invited to participate in the immunobridging substudy at the time of enrolment; all women were invited until the target enrolment of 910 participants was reached.

The primary efficacy analysis was at month 18, with the final analysis at month 36 evaluating durability. Vaccine efficacy of the 2-valent vaccine against incident persistent HPV16 and HPV18 infection was 97·5% (95% CI 81·7–99·7) at month 18 and 97·5% (90·0–99·4) at month 36.^7^, ^19^ 9-valent vaccine efficacy at the same timepoints was 97·5% (95% CI 81·6–99·7) and 98·8% (91·3–99·8), respectively.

### Sample selection

The immunobridging study used blood samples from all girls in the one dose groups in the DoRIS trial who attended the month 24 visit within a window of 22–28-months after vaccination. For the KEN SHE trial, we took a random sample of 155 participants in the immunobridging substudy in each HPV vaccine group, from those who had blood samples available from month 0 and month 24, and the month 24 sample was taken within the 22–28-month window.

### Laboratory methods

Antibodies to HPV16 and HPV18 were measured by type-specific virus-like particle ELISA assay at the Frederick National Laboratory for Cancer Research HPV Immunology Laboratory in Frederick, MD, USA (appendix 2 p 2). Antibody concentrations greater than or equal to the lower limit of detection for each assay were pre-specified to indicate seropositivity (HPV16 ≥1·309 international units [IU]/mL and HPV18 ≥1·109 IU/mL). HPV DNA genotyping was done using Anyplex II HPV28 (Seegene, Seoul, South Korea) at the Catalan Institute of Oncology, Barcelona, Spain (DoRIS) and the University of Washington East Africa STI Laboratory, Mombasa, Kenya (KEN SHE).

### Sample size

Our sample size calculations were based on an expected 130 girls in each group in the DoRIS trial available for the per-protocol analysis at month 24, assuming a 20% loss to follow-up across 36 months and 5% HPV seropositive or DNA positive at enrolment in the DoRIS trial. With 130 in each group from each trial, if the true GMC ratio (DoRIS:KEN SHE) between groups is 1·0, we had more than 90% power to show that the lower limit of the 95% CI for the GMC ratio was above 0·50, indicating that the one dose schedule in DoRIS did not decrease HPV16 and HPV18 antibody GMC by more than 50% compared with KEN SHE. The non-inferiority margin of 0·50 was defined a priori on the basis of that used in other HPV vaccine trials.^20^, ^21^ We assumed an SD of 0·50–0·60 log_10_ anti-HPV concentration,^20^, ^22^ and used a one-sided non-inferiority test at the 2·5% level.

### Statistical analysis

The primary immunobridging analysis was in the per-protocol cohort: participants who received one dose of HPV vaccine and who were HPV antibody negative and DNA negative at enrolment for the genotype under analysis. Secondary analyses included all participants who received one dose, irrespective of baseline antibody or HPV DNA status.

Separate analyses were done for each vaccine type. HPV antibody concentrations were log_10_-transformed; concentrations less than the assay cutoff were given a value of half the cutoff before log transformation. Arithmetic mean log_10_ antibody concentrations and 95% CIs were calculated for each group, assuming a normal distribution.

The difference in HPV genotype-specific log_10_ concentrations at month 24 between the two groups (DoRIS minus KEN SHE) and its 95% CI were calculated; the GMC ratio and its 95% CI were obtained by back-transformation. Non-inferiority of the antibody response was concluded if the lower bound for the two-sided 95% CI for the GMC ratio was above 0·50. Linear regression with a fixed term for the study groups was used to obtain p values; p values less than 0·05 were considered statistically significant.

The number and proportion of participants in each group who were seropositive for HPV16-specific and HPV18-specific antibodies at month 24 was tabulated. Seropositivity for a particular HPV genotype was defined as an antibody level higher than the assay cutoff. For each vaccine and HPV genotype, we calculated the difference (one dose of DoRIS minus one dose of KEN SHE) in the proportion of seropositive individuals and estimated the 95% CI for the difference using the exact method of Chan and Zhang.^23^ Non-inferiority of seropositivity was concluded if the lower bound of the two-sided 95% CI for the difference was higher than –5%.

For the primary outcomes, success was required for both HPV16 and HPV18 to conclude non-inferiority for each vaccine; therefore, no multiplicity adjustment was made to account for the testing of multiple HPV genotypes. Missing data were minimal (<1%) so a complete case analysis was used. SAS (version 9.1) and Stata (version 17) were used for all analyses.

### Role of the funding source

The funders of this study did not have any role in the study design, data collection, data analysis, data interpretation, or writing of the report.

## Results

The DoRIS trial enrolled 930 participants (155 per group); 154 (99%) participants in the one dose 2-valent vaccine group and 152 (98%) in the one dose 9-valent vaccine group attended the month 24 visit within the 22–28-month timeframe so were included in the immunobridging analysis. The KEN SHE trial enrolled 302 participants in the immunobridging substudy in the one dose 2-valent vaccine group, and 303 in the one dose 9-valent vaccine group. Of those, 287 (95%) and 278 (92%), respectively, attended the month 24 visit within the required timeframe; 154 were randomly sampled from each group.

Owing to protocol-specified differences in eligibility requirements, DoRIS participants were younger (median [IQR] 10 years [9–12]) than those in KEN SHE (18·5 years [17–19]; table 1). In addition, baseline HPV16 and HPV18 seropositivity and DNA positivity was higher in KEN SHE than in DoRIS, consistent with the older age range and that all KEN SHE participants were sexually active. Therefore a larger proportion of KEN SHE participants than DoRIS participants were excluded from the per-protocol analyses.

**Table 1:** Demographic characteristics at baseline among one dose groups in the DoRIS and KEN SHE trials

		**One dose DoRIS (Cervarix; n=154)**	**One dose KEN SHE (Cervarix; n=154)**	**One dose DoRIS (Gardasil-9; n=152)**	**One dose KEN SHE (Gardasil-9; n=154)**
Age, years		10 (9–12)	18·5 (17–19)	10 (9–12)	18 (17–19)
Age group, years					
	9–14	154 (100%)	0	152 (100%)	0
	15–20	0	154 (100%)	0	154 (100%)
HPV16 seropositive at baseline		6 (4%)	40 (26%)	7 (5%)	25 (16%)
HPV18 seropositive at baseline		13 (8%)	49 (32%)	16 (11%)	30 (19%)
HPV16 DNA positive (cervical) at baseline		0	14 (9%)	1 (<1%)	9 (6%)
HPV18 DNA positive (cervical) at baseline		0	6 (4%)	1 (<1%)	3 (2%)
In per-protocol analysis					
	HPV16*	148 (96%)	109 (71%)	145 (95%)	121 (79%)
	HPV18*	141 (92%)	99 (64%)	136 (89%)	123 (80%)
	Age, years†	10 (9–12)	19 (17–19)	10 (9–12)	18 (17–19)

Data are n (%) or median (IQR). HPV=human papillomavirus.

* Numbers in the per-protocol analysis for each HPV genotype.

† Median (IQR) age in the per-protocol population does not differ between the subgroup for the HPV16 analysis and that for the HPV18 analysis, for any of the four groups.

**Figure 1:**
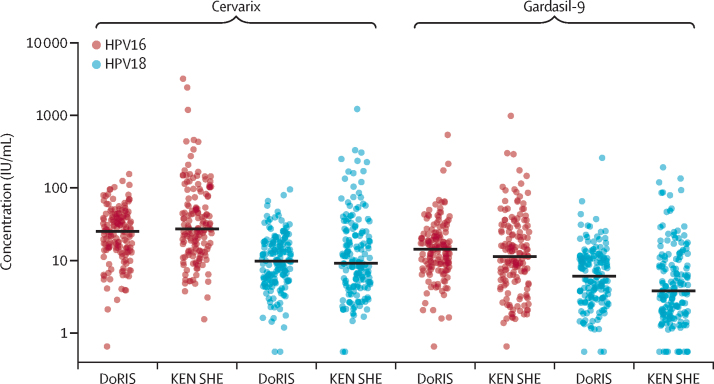
Distribution of HPV16 and HPV18 antibody concentrations at 24 months after a single dose of HPV vaccine, by group (total vaccinated population) Each data point represents a single individual and the line through the data points represents the median concentration. HPV=human papillomavirus. IU=international unit.

**Figure 2:**
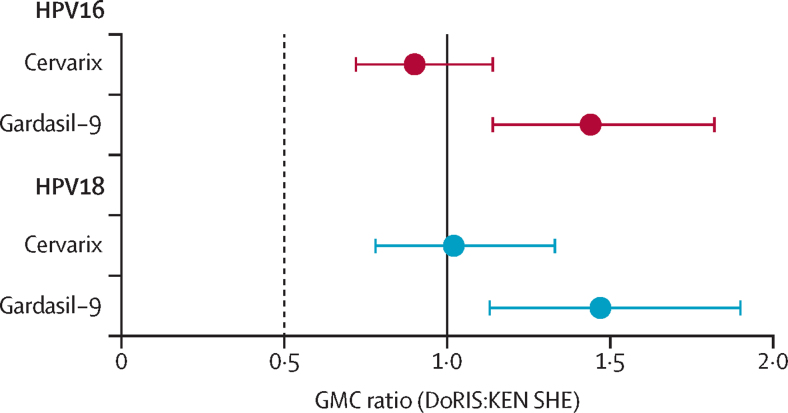
Ratio of GMCs (DoRIS:KEN SHE) and 95% CIs, at 24 months after a single dose of HPV vaccine (per-protocol population) The black dotted line represents the non-inferiority margin. The solid black line is a GMC ratio of 1 (ie, no difference between the two groups). GMC=geometric mean concentration. HPV=human papillomavirus.

In the per-protocol analysis of the 2-valent vaccine, 147 (99%) of 148 participants in the DoRIS trial and 109 (100%) of 109 participants in the KEN SHE trial were seropositive for IgG antibodies to HPV16, and 139 (99%) of 141 and 97 (98%) of 99, respectively, for HPV18 (table 2). HPV16 and HPV18 antibody GMCs after one dose of the 2-valent vaccine were similar in DoRIS (p=0·39) and in KEN SHE (p=0·91; figure 1). Non-inferiority of antibody concentrations for the 2-valent vaccine was met for both HPV genotypes, with GMC ratios (DoRIS:KEN SHE) of 0·90 (95% CI 0·72 to 1·14) for HPV16 and 1·02 (0·78 to 1·33) for HPV18 (figure 2). Non-inferiority was also met for seropositivity, with a difference of −0·7% (95% CI −3·9 to 3·0) for HPV16 and 0·1% (−3·2 to 4·1) for HPV18.

**Table 2:** Comparisons of GMCs and seropositivity rates at month 24 post-single dose HPV vaccination between DoRIS and KEN SHE (per-protocol population)*

	N*	**GMC**†**(95% CI), IU/mL**	**IQR**	**Seropositive**‡**n (%)**
**HPV16 IgG antibody**				
DoRIS (Cervarix)	148	22·9 (19·9 to 26·4)	14·7 to 40·0	147 (99%)
KEN SHE (Cervarix)	109	25·3 (21·0 to 30·6)	13·0 to 43·2	109 (100%)
GMC ratio, DoRIS:KEN SHE (95% CI)	..	..	0·90 (0·72 to 1·14)	..
Difference in seropositive, DoRIS–KEN SHE (95% CI)	..	..	−0·7% (−3·9 to 3·0)	..
DoRIS (Gardasil-9)	145	13·7 (11·9 to 15·8)	8·9 to 21·4	144 (99%)
KEN SHE (Gardasil-9)	121	9·5 (7·8 to 11·5)	4·8 to 19·1	120 (99%)
GMC ratio, DoRIS:KEN SHE (95% CI)	..	..	1·44 (1·14 to 1·82)	..
Difference in seropositive, DoRIS–KEN SHE (95% CI)	..	..	0·1% (−3·2 to 4·1)	..
**HPV18 IgG antibody**				
DoRIS (Cervarix)	141	9·9 (8·5 to 11·5)	5·7 to 17·7	139 (99%)
KEN SHE (Cervarix)	99	9·7 (7·6 to 12·4)	4·3 to 21·8	97 (98%)
GMC ratio, DoRIS:KEN SHE (95% CI)	..	..	1·02 (0·78 to 1·33)	..
Difference in seropositive, DoRIS–KEN SHE (95% CI)	..	..	0·6% (−3·5 to 6·0)	..
DoRIS (Gardasil-9)	136	5·7 (4·9 to 6·8)	3·0 to 10·8	133 (98%)
KEN SHE (Gardasil-9)	123	3·9 (3·2 to 4·8)	1·8 to 8·4	113 (92%)
GMC ratio, DoRIS:KEN SHE (95% CI)	..	..	1·47 (1·13 to 1·90)	..
Difference in seropositive, DoRIS–KEN SHE (95% CI)	..	..	5·9% (0·5 to 12·5)	..

GMC=geometric mean concentration. HPV=human papillomavirus. IU=international unit.

* DoRIS and KEN SHE participants who were ELISA antibody negative and DNA negative at baseline (pre-vaccination) for the HPV genotype under analysis.

† ELISA serum antibody GMC.

‡ Seropositivity defined as concentrations greater than the laboratory determined cutoff (HPV16 1·309 IU/mL and HPV18 1·109 IU/mL).

In the per-protocol analysis of the 9-valent vaccine, 144 (99%) of 145 participants in the DoRIS trial and 120 (99%) of 121 participants in the KEN SHE trial, were seropositive for IgG antibodies to HPV16, and 133 (98%) of 136 and 113 (92%) of 123 for HPV18 (table 2). HPV16 and HPV18 antibody GMCs after one dose of the 9-valent vaccine were significantly higher in DoRIS than in KEN SHE (p=0·002 and p=0·004, respectively). Non-inferiority of antibody concentrations was met for the 9-valent vaccine for both HPV genotypes, with GMC ratios of 1·44 (95% CI 1·14 to 1·82) for HPV16, and 1·47 (1·13 to 1·90) for HPV18 (figure 2). Non-inferiority was also met for seropositivity, with a difference of 0·1% (95% CI −3·2 to 4·1) for HPV16 and 5·9% (0·5 to 12·5) for HPV18.

In the total vaccinated cohort, non-inferiority of antibody GMCs and seropositivity was shown for the 2-valent and 9-valent vaccines, for both HPV genotypes (table 3).

**Table 3:** Comparisons of GMC and seropositivity rates at month 24 post-single dose HPV vaccination between DoRIS and KEN SHE (total vaccinated population)*

	N*	**GMC**†**(95% CI), IU/mL**	**IQR**	**Seropositive**‡**n (%)**	**Seroconverted**§**n (%)**
**HPV16 IgG antibody**					
DoRIS (Cervarix)	154	22·7 (19·8 to 26·1)	14·5 to 39·8	153 (99%)	147 (95%)
KEN SHE (Cervarix)	154	32·3 (26·5 to 39·3)	13·5 to 71·1	154 (100%)	114 (74%)
GMC ratio, DoRIS:KEN SHE (95% CI)	..	..	0·70 (0·55 to 0·89)	..	..
Difference in seropositive, DoRIS–KEN SHE (95% CI)	..	..	−0·6% (−3·7 to 1·8)	..	..
DoRIS (Gardasil-9)	152	14·1 (12·2 to 16·3)	8·8 to 21·8	151 (99%)	144 (95%)
KEN SHE (Gardasil-9)	154	12·3 (10·1 to 14·9)	5·4 to 28·4	153 (99%)	128 (83%)
GMC ratio, DoRIS:KEN SHE (95% CI)	..	..	1·15 (0·90 to 1·46)	..	..
Difference in seropositive, DoRIS–KEN SHE (95% CI)	..	..	0 (−3·1 to 3·0)	..	..
**HPV18 IgG antibody**					
DoRIS (Cervarix)	154	9·6 (8·3 to 11·1)	5·5 to 17·5	152 (99%)	139 (90%)
KEN SHE (Cervarix)	154	11·8 (9·5 to 14·6)	4·7 to 25·2	152 (99%)	102 (66%)
GMC ratio DoRIS:KEN SHE (95% CI)	..	..	0·81 (0·63 to 1·05)	..	..
Difference in seropositive, DoRIS–KEN SHE (95% CI)	..	..	0 (−3·5 to 3·5)	..	..
DoRIS (Gardasil-9)	152	6·0 (5·2 to 7·0)	3·0 to 10·9	149 (98%)	133 (88%)
KEN SHE (Gardasil-9)	154	4·7 (3·9 to 5·8)	1·9 to 10·5	144 (94%)	114 (74%)
GMC ratio, DoRIS:KEN SHE (95% CI)	..	..	1·27 (0·99 to 1·64)	..	..
Difference in seropositive, DoRIS–KEN SHE (95% CI)	..	..	4·5% (−0·1 to 9·9)	..	..

GMC=geometric mean concentration. HPV=human papillomavirus. IU=international unit.

* All participants (irrespective of ELISA antibody or HPV DNA status at baseline).

† ELISA serum antibody GMC.

‡ Seropositivity defined by the laboratory determined cutoff (HPV16 1·309 IU/mL and HPV18 1·109 IU/mL).

§ Seroconversion defined as concentrations greater than the laboratory determined cutoff among participants who were seronegative for the HPV genotype at baseline.

## Discussion

We found that immune responses 24 months after a single dose of two different HPV vaccines in girls in the target age range for vaccination were non-inferior to those in young women in Kenya who were randomly assigned to a single dose of the same vaccine and in whom efficacy was shown. These results from the first two randomised trials of the single-dose schedule provide the strongest available evidence that one dose of HPV vaccine induces immune responses in young girls that are comparable to those seen in young women in whom efficacy has been shown. In April, 2022, WHO's Strategic Advisory Group of Experts (SAGE) recommended that HPV vaccine dose schedules be updated to allow countries to choose a one-dose or two-dose schedule for individuals aged 9–20 years.^24^ In December, 2022, WHO published a new position paper, stating that a single-dose schedule can provide comparable efficacy and durability of protection as two doses and confirming the recommendations of SAGE.^11^

The SAGE recommendations were based in part on evidence from the KEN SHE and DoRIS trials. These trials are particularly important because they were conducted in a region with one of the highest HPV infection and cervical cancer rates in the world. In the control group of KEN SHE, the incidence of persistent infection with 9-valent HPV vaccine genotypes at month 18 (9·2 per 100 person-years) was around 30% higher than has been reported in other trials.^19^ The HPV16 and HPV18 vaccine efficacy of higher than 97% observed in the KEN SHE trial is comparable to the three-dose efficacy observed in several licensure trials, and provides strong evidence for single-dose protection. The results of the DoRIS trial provide further insight on vaccine-induced antibodies in young girls up to 36 months after vaccination with a single dose.^9^

Immunobridging studies provide a valuable strategy to evaluate reduced-dose schedules in young girls, the primary target group for HPV vaccination but in whom evaluating efficacy is difficult because of the time needed to accrue virological or disease endpoints. In immunobridging studies, HPV genotype-specific antibody concentrations in a new population group are compared with those in a population for which efficacy has been shown; if antibody concentrations are shown to be non-inferior, then efficacy is also assumed to be comparable. This approach was taken for the original licensure of HPV vaccines in girls aged 9–14 years, and the approval of a two-dose schedule in this age range.^25^, ^26^ Antibody concentrations are a recommended endpoint for immunobridging because protection conferred by virus-like particle HPV vaccines is considered to be mediated primarily by neutralising antibodies.^27^ Total IgG as measured by the ELISA assay has been shown to correlate well with HPV16 and HPV18 neutralisation assays, even at the lower antibody concentrations observed after a single dose.^28^ However, the minimum antibody concentration needed for protection has not been established.

Age is a key determinant of antibody responses following HPV vaccination, with young girls having significantly higher antibody GMCs than young women. Notably, HPV16 and HPV18 GMCs after a single dose of the 2-valent vaccine in DoRIS were similar or lower to those in KEN SHE, despite the older age in KEN SHE (median age 18·5 years *vs* 10 years). This was particularly pronounced in the total vaccinated population, for which HPV16 antibody concentrations in DoRIS were significantly lower than those in KEN SHE. These findings are similar to those in our immunobridging study of DoRIS with CVT, in which antibody GMCs in the total vaccinated population after one dose of the 2-valent vaccine were lower (although not statistically significant) in DoRIS than CVT, despite the older age in CVT (median age 20 years).^10^ One explanation could be that vaccination boosts an individual's response to previous natural infection, or that boosting of vaccine antibody responses occurred during subsequent sexual exposure to HPV. The KEN SHE participants were all sexually active and 38% had evidence of current or previous HPV16 and HPV18 infection at enrolment. In contrast, only 1·5% of all DoRIS participants reported having passed sexual debut by month 24, and 11% had any evidence of HPV16 and HPV18 infection at enrolment. In the IARC/India trial (girls aged 10–18 years), a small increase in HPV16 and HPV18 antibody GMCs in the single-dose recipients was noted between month 36 and month 120; the authors speculated that this might have reflected a boosting effect as girls became sexually active.^17^ In contrast, HPV16 and HPV18 antibody GMCs after a single dose of 9-valent vaccine were higher in DoRIS than in KEN SHE, even in the total vaccinated population, consistent with their younger age. The reasons for these differences in age effect between vaccines are unknown; however, a higher proportion of 2-valent than 9-valent recipients in KEN SHE had evidence of HPV infection at baseline (45% *vs* 30%, p<0·01). A further explanation is that the potent adjuvant of the 2-valent vaccine^22^ could over-ride the age effect seen after one dose of the 9-valent vaccine.

In both trials, HPV16 and HPV18 antibody GMCs were higher after the 2-valent than the 9-valent vaccine. These results are similar to other studies that compared the 2-valent and 4-valent vaccines.^22^, ^29^ Despite this, both vaccines have similar high efficacy against persistent HPV16 and HPV18 infection and disease. HPV18 antibody GMCs and seropositivity were lower than those for HPV16, for both vaccines. This finding has been reported in other studies, despite high clinical efficacy against HPV18-related persistent infection and related sequelae.^20^, ^28^, ^30^

Strengths of our study include the immunobridging of results from two different HPV vaccines in girls in the target age range for vaccination to those from the first randomised trial of the efficacy of the single-dose regimen. We bridged our immune responses at 24 months after vaccination, after the one dose antibody concentrations had reached a plateau. Both trials were conducted in a region with extremely high HPV infection and cervical cancer rates, and where vaccination is most needed. The trials were also conducted in areas where malaria is endemic and has the potential to affect the immune response. We tested the samples from both trials in the same laboratory, using the same internationally standardised and well validated assay, allowing reproducibility of results and comparison with other studies. Both trials had excellent retention at the relevant visits for this study.

Limitations of our study include that it was restricted to participants who were HIV negative. The efficacy of reduced dose HPV vaccine regimens in women living with HIV is still uncertain, and WHO continues to recommend that immunocompromised individuals receive three doses when possible.^11^ Another important question is whether a single dose would provide protection in women who are vaccinated at ages older than 20 years, and among those who are infected with HPV vaccine genotypes. Although data from national HPV vaccination programmes have shown some effectiveness of one dose in women vaccinated up to the age of 30 years, the highest effectiveness is in younger age groups.^31^ Estimates of effectiveness vary depending on whether the analysis includes a buffer period (lag time) to allow prevalent HPV infections to clear, which is likely to be of greater importance when evaluating the single-dose regimen in older individuals.^31^

Nigeria introduced HPV vaccination in October, 2023, with a single-dose regimen, the first African country to do so, and Tanzania will switch to single-dose delivery in 2024. Although the single-dose schedule has the potential to greatly increase vaccine coverage, the vaccine effectiveness at the population level is unlikely to equal the greater than 97% efficacy seen in KEN SHE. A recent study in Rwanda estimated vaccine effectiveness of 70% among vaccinated women aged 17–29 years; the authors suggested this finding was likely because some women were already sexually active when vaccinated.^32^

Of note, our immunobridging study is based on only 24 months of follow-up, and our data on durability of the immune response is only up to 36 months. Since antibody concentrations in DoRIS were stable between month 24 and month 36,^9^ it is likely that immune responses would be non-inferior to those in KEN SHE at month 36, for which efficacy was also shown. We are continuing follow-up of the DoRIS trial cohort to 9 years post-vaccination, which will provide further information on long-term immune responses in young girls. When comparing GMCs, we used a pre-specified non-inferiority margin of 0·50. Although a more stringent margin of 0·67 was met for the per-protocol population, it was not met for the 2-valent vaccine in the total vaccinated population. Since an immune correlate of protection is undefined, the significance of this finding is unclear. Lastly, the per-protocol population excluded a large proportion of the KEN SHE participants, because of previous or current HPV infection at baseline, although the pre-specified non-inferiority margins were still met when these women were included.

In summary, our immunobridging results provide evidence that one dose of HPV vaccine in young girls induces antibody concentrations 24 months after vaccination that could be protective against persistent HPV16 and HPV18 infection. A single-dose HPV vaccine schedule could substantially reduce the costs and logistical challenges of vaccine delivery, alleviate vaccine supply constraints, and expand global vaccine introductions and coverage.

## Equitable partnership declaration

## Data sharing

De-identified participant data presented in this manuscript can be made available after publication following written request to the London School of Hygiene & Tropical Medicine and the Mwanza Intervention Trials Unit, Tanzania. Requests must be accompanied by an analysis plan, which will be reviewed by the Mwanza Intervention Trials Unit Data Sharing Committee and lead investigators for each trial. Requesting researchers will be required to sign a Data Access Agreement if approval is given.

## Declaration of interests

KB reports grants from the Bill & Melinda Gates Foundation during the conduct of the DoRIS trial, and a grant and vaccine donations from Merck Pharmaceuticals outside the submitted work. DW-J reports grants from the Gates Foundation and the UK Research and Innovation Medical Research Council during the DoRIS trial, and a grant and vaccine donations from Merck outside the submitted work. HW reports grants from the Gates Foundation and the UK Research and Innovation Medical Research Council during the DoRIS trial, and vaccine donations from Merck outside the submitted work. JC reports grant support from the Gates Foundation and the UK Research and Innovation Medical Research Council during the DoRIS trial. RJH reports a grant from the UK Research and Innovation Medical Research Council during the DoRIS trial. RVB reports grants from the Gates Foundation during the conduct of the KEN SHE trial, and data monitoring committee honorarium from Gilead Sciences and manuscript and abstract writing support from Regeneron Pharmaceuticals outside the submitted work. NRM reports grant support from Merck Pharmaceuticals outside the submitted work. DAG reports grants from the Gates Foundation during the conduct of the KEN SHE trial, and grants and personal fees from Merck outside the submitted work. EAB reports grants from the National Institutes of Health, the Centers for Disease Control and Prevention, and the European and Developing Countries Clinical Trials Partnership during the conduct of the KEN SHE trial; and personal fees from Gilead Sciences and personal fees from Merck outside the submitted work. All other authors declare no competing interests.

## Supplementary Materials

KiSwahili translation of the abstract

Supplementary appendix 2

Equitable Partnership Declaration
