# Supplementary material for: Comparing one dose of HPV vaccine in girls aged 9–14 years in Tanzania (DoRIS) with one dose in young women aged 15–20 years in Kenya (KEN SHE): an immunobridging analysis of randomised controlled trials
Source: Lancet Glob Health. 2024 Feb 14;12(3):e491–9. doi: 10.1016/S2214-109X(23)00586-7 (PMC10882205; doi:10.1016/S2214-109X(23)00586-7)
Supplement: KiSwahili translation of the abstract [file mmc1.pdf]

THE LANCET
Global Health

Supplementary appendix 1

This translation in Kiswahili was submitted by the authors and we reproduce it as supplied. It has not been peer reviewed. *The Lancet*'s editorial processes have only been applied to the original in English, which should serve as reference for this manuscript.

Tafsiri hii katika Kiswahili iliwasilishwa na waandishi na tunatengeneza tena kama hutolewa. Haijapitiwa. Mchakato wa hariri wa *Lancet* umetumika tu kwa asili kwa Kiingereza, ambayo inapaswa kutumika kama kumbukumbu kwa muswada hii.

Supplement to: Baisley K, Kemp TJ, Mugo NR, et al. Comparing one dose of HPV vaccine in girls aged 9–14 years in Tanzania (DoRIS) with one dose in young women aged 15–20 years in Kenya (KEN SHE): an immunobridging analysis of randomised controlled trials. *Lancet Glob Health* 2024; **12:** e491–99.

Kulinganisha kinga mwili iliyopatikana baada ya dozi moja ya chanjo ya HPV kwa wasichana wa umri wa miaka 9 hadi 14 katika utafiti wa DoRIS nchini Tanzania na iliyopatikana baada ya dozi moja kwa wasichana wenye umri wa miaka 15 hadi 20 katika utafiti wa KEN SHE nchini Kenya

Muhtasari

Utangulizi

Utafiti wa kwanza wa majaribio wa ufanisi wa dozi moja ya chanjo ya HPV uitwao 'KEN SHE' ulionyesha ufanisi wa zaidi ya asilimia 97 katika kuzuia maambukizi yasiyoondolewa ya virusi vya HPV16 na HPV 18 miezi 36 baada ya chanjo kutolewa kwa wanawake nchini Kenya. Tumelinganisha mwitikio wa kingamwili baada ya dozi moja ya chanjo ya HPV katika utafiti wa DoRIS, utafiti wa kwanza wa dozi moja kwa wasichana wenye umri wa miaka 9 – 14, umri unaolengwa katika mpango wa kutoa chanjo ya HPV, na mwitikio wa kingamwili uliopatikana baada ya dozi moja ya chanjo ya aina hiyo hiyo katika utafiti wa KEN SHE.

Mbinu zilizotumika katika utafiti

Katika utafiti wa DoRIS, wasichana 930 wenye umri wa miaka 9 - 14, nchini Tanzania walipangwa katika makundi kwa njia ya bahati nasibu na kupata dozi moja, mbili au tatu za chanjo inayozuia aina mbili za virusi vya HPV (Cervarix) au chanjo inayozuia aina tisa za virusi vya HPV (Gardasil). Idadi ya waliotengeneza kingamwili na kiwango cha kinga mwilini miezi 24 baada ya kutolewa chanjo dozi moja ililinganishwa na ile ya wanawake wenye umri wa miaka 15 - 20 waliopangwa kwa njia ya bahati nasibu na kupewa dozi moja ya chanjo ile ile katika utafiti wa KEN SHE. Sampuli ziliwekwa katika mafungu na kupimwa kwa pamoja kwa njia ya ELISA ili kutambua sampuli ambazo zina kinga aina ya IgG dhidi ya virusi vya HPV16 na HPV18. Uwiano wa kiwango cha kinga kati ya DoRIS na KEN SHE ili usiwe duni ulipangwa kuwa kikomo cha chini kwa asilimia 95 kisiwe chini ya 0.50

Matokeo ya utafiti

Kiwango cha kinga dhidi ya HPV 16 na HPV 18 miezi 24 tangu kutolewa kwa dozi moja katika utafiti wa DoRIS kilifanana au kuwa juu ya kiwango cha kinga katika utafiti wa KEN SHE. Uwiano wa kiwango cha kinga kwa chanjo inayozuia virusi aina mbili (2-valent) ulikuwa 0.9 (95% CI 0.72 -1.14) kwa HPV 16 na 1.02 (0.78 – 1.33) kwa HPV 18. Uwiano wa kiwango cha kinga kwa chanjo inayozuia virusi aina tisa (9-valent) ulikuwa 1.44 (95% CI 1.14 – 1.82) kwa

HPV 16 na 1.47 (1.13 -1.90) kwa HPV 18. Utengenezaji wa kinga na kiwango cha kinga dhidi ya HPV 16 na HPV 18 katika utafiti wa DoRIS ikilinganishwa na ule wa KEN SHE haukuwa hafifu na ulifikia kiwango kilichowekwa kwa chanjo zote.

Tafsiri ya matokeo ya utafiti huu

Mwitikio wa kinga dhidi ya virusi vya HPV16 na HPV18 kwa wasichana wadogo miezi 24 baada ya dozi moja ya chanjo inayozuia virusi aina mbili au tisa ilikuwa sawa na ule uliopatikana kwa vijana wa kike waliopangwa kupata dozi moja ya chanjo ile ile kwa njia ya bahati nasibu na kuonyesha ufanisi. Dozi moja ya chanjo ya HPV ikitolewa kwa wasichana walio na umri uliolengwa katika utoaji wa chanjo, inasababisha mwitikio wa kinga ambao unaweza kuwa na uwezo wa kuzuia maambukizi ya HPV16 na HPV 18 yasiyoondolewa na kingamwili.
