# Supplementary material for: Comparing one dose of HPV vaccine in girls aged 9–14 years in Tanzania (DoRIS) with one dose in young women aged 15–20 years in Kenya (KEN SHE): an immunobridging analysis of randomised controlled trials
Source: Lancet Glob Health. 2024 Feb 14;12(3):e491–9. doi: 10.1016/S2214-109X(23)00586-7 (PMC10882205; doi:10.1016/S2214-109X(23)00586-7)
Supplement: Supplementary appendix 2 [file mmc2.pdf]

THE LANCET
Global Health

## Supplementary appendix 2

This appendix formed part of the original submission and has been peer reviewed. We post it as supplied by the authors.

Supplement to: Baisley K, Kemp TJ, Mugo NR, et al. Comparing one dose of HPV vaccine in girls aged 9–14 years in Tanzania (DoRIS) with one dose in young women aged 15–20 years in Kenya (KEN SHE): an immunobridging analysis of randomised controlled trials. *Lancet Glob Health* 2024; **12:** e491–99.

## Supplementary appendix

## Table of Contents

Methods ........ 1
DoRIS trial ........ 1
The KEN SHE trial ........ 1
Laboratory methods ........ 2
References ........ 3

## Methods

### DoRIS trial

The DoRIS trial [Dose Reduction Immunobridging and Safety Study of two HPV vaccines in Tanzanian Girls; clinicaltrials.gov: NCT02834637] is the first randomised trial to evaluate immunogenicity of the single dose regimen in girls in the target age range for vaccination. The trial commenced enrolment in February 2017 and has been described in detail previously.[1] In brief, it is an ongoing unblinded, individually-randomised controlled trial comparing 1, 2 and 3 doses of two HPV vaccines among 930 healthy, HIV-negative Tanzanian schoolgirls aged 9-14 years. The trial is being conducted in Mwanza, in the Lake Victoria zone of north-western Tanzania. Girls were randomly allocated to one of 6 arms comprising 3 different dose schedules of the GSK 2-valent or Merck 9-valent vaccine: the originally recommended 3 dose schedule given at 0, 1 and 6 months (2-valent vaccine) or 0, 2 and 6 months (9-valent); 2 doses given 6 months apart; or a single dose. Before vaccination, girls were asked to collect a vaginal swab which was used to detect HPV DNA. Blood samples for HPV immune responses including IgG antibodies to HPV 16/18, antibody avidity and memory B cell responses were taken at baseline, Month (M) 1, M7, M12, M24 and M36. All participants were followed up to M36 for safety and immunogenicity evaluations. Girls in the 1 and 2 dose arms have been enrolled in a trial extension and will be followed up to 9 years (M108).

The trial was approved by the Tanzanian Medical Research Coordinating Committee and the ethics committee of the London School of Hygiene and Tropical Medicine. Written informed consent was obtained from parents/guardians, with written assent from participants.

### The KEN SHE trial

KEN SHE (NCT03675256) is the first randomised controlled efficacy trial of a single dose of HPV vaccine. The trial enrolled 2275 sexually-active young women aged 15-20 years from 3 sites in Kenya (Thika, Nairobi and Kisumu) between December 2018 and November 2019.[2] Women were randomly allocated to one of 3 arms, comprising a single dose of the GSK 2-valent HPV vaccine (N=760), the Merck 9-valent HPV vaccine (N=758), or meningococcal vaccine (N=757). Women were seen at M3, M6 and then 6-monthly for 36 months; cervical swabs for HPV DNA testing were collected at each visit. Women enrolled in the main trial were invited to participate in the immunobridging sub-study at the time of enrolment; all

women were invited until the target enrolment of 910 participants was reached. Blood samples for immunogenicity were collected at enrolment, M1 and M24.

The trial was approved by the Kenya Medical Research Institute (KEMRI) Scientific and Ethics Review Unit (SERU) and the University of Washington (UW) Institutional Review Board (IRB).

**Laboratory methods**

Antibodies to HPV-16 and HPV-18 were measured by type-specific virus like particles (VLP) ELISA assay at the Frederick National Laboratory for Cancer Research HPV Immunology Laboratory in Maryland, USA. The DoRIS samples were originally tested at the Frederick laboratory in March 2021. For the immunobridging study, samples from the KEN SHE trial and a 20% simple random sample of the M24 DoRIS trial samples (30 per arm) were batched and tested together in April 2022 in the same laboratory, using the same assays and procedures as in 2021. The re-test results for the DoRIS samples were evaluated for between-run acceptability; concentrations were required to be within ±20% of the original results for concentrations >20 EU/mL, or within 25% of the original results, for concentrations ≤20 EU/mL. We required ≥80% of the re-test results to meet the acceptability criteria, or all DoRIS samples were to be retested. The acceptability criteria were pre-defined beforehand, and were based on United States Food and Drug Administration (US FDA) recommendations.[3] During ELISA testing, laboratory staff were blinded to the trial, HPV vaccine dose group and timepoint of the samples. Antibody concentrations greater than or equal to the lower limit of detection for each assay were pre-specified to indicate seropositivity (HPV16: ≥1.309 international units [IU]/mL; HPV18: ≥1.109 IU/mL).

The difference in antibody concentration between the original test and retest for the DoRIS samples was <20% in 88.3% of samples for HPV16 and 94.8% of samples for HPV18. Therefore, the retest results met the acceptability criteria and no further retesting was done. The original DoRIS results were used in the analyses.

In the DoRIS trial, HPV DNA genotyping at enrolment was done using the Anyplex II HPV28 (Seegene, South Korea), a multiplex, type-specific, real-time PCR-based detection assay, at the Catalan Institute of Oncology, Barcelona. In KEN SHE, HPV DNA genotyping was conducted using the same assay at the University of Washington East Africa STI Laboratory, Mombasa, Kenya.

## References

1. Baisley KJ, Whitworth HS, Changalucha J, et al. A dose-reduction HPV vaccine immunobridging trial of two HPV vaccines among adolescent girls in Tanzania (the DoRIS trial) - Study protocol for a randomised controlled trial. Contemp Clin Trials 2021; 101: 106266

2. Barnabas RV, Brown ER, Onono M, et al.; KEN SHE Study Team. Single-dose HPV vaccination efficacy among adolescent girls and young women in Kenya (the KEN SHE Study): study protocol for a randomized controlled trial. Trials. 2021 Sep 27;22(1):661.

3. https://www.fda.gov/regulatory-information/search-fda-guidance-documents/bioanalytical-method-validation-guidance-industry
