# Supplementary material for: Comparing one dose of HPV vaccine in girls aged 9–14 years in Tanzania (DoRIS) with one dose in young women aged 15–20 years in Kenya (KEN SHE): an immunobridging analysis of randomised controlled trials
Source: Lancet Glob Health. 2024 Feb 14;12(3):e491–9. doi: 10.1016/S2214-109X(23)00586-7 (PMC10882205; doi:10.1016/S2214-109X(23)00586-7)
Supplement: Equitable Partnership Declaration [file mmc3.pdf]

THE LANCET
Global Health

Supplementary appendix 3

This Equitable Partnership Declaration (EPD) was submitted by the authors, and we reproduce it as supplied. It has not been peer reviewed. *The Lancet*'s editorial processes have not been applied to the EPD.

Supplement to: Baisley K, Kemp TJ, Mugo NR, et al. Comparing one dose of HPV vaccine in girls aged 9–14 years in Tanzania (DoRIS) with one dose in young women aged 15–20 years in Kenya (KEN SHE): an immunobridging analysis of randomised controlled trials. *Lancet Glob Health* 2024; **12:** e491–99.

Equitable Partnership Declaration questions

This Equitable Partnership Declaration is a statement being published online alongside papers at *The Lancet Global Health*, as a separate appendix, to allow researchers to describe how their work engages with researchers, communities, and environments in the countries of study. This is part of our broader goal to decolonise global health, handing control and leadership of research to academics and clinicians who are based in the regions of study, and to affected communities.

Please answer all questions with as much detail as possible, noting that all included information will be published open-access and it will be freely available online to all who wish to read it. If a question does not apply to your study, please state "Not applicable".

The format of and questions in this statement are currently in a pilot phase. Please email Dr Liam Messin (Liam.Messin@lancet.com; deputy editor) and Dr Kate McIntosh (Kate.McIntosh@lancet.com; senior editor) with any feedback, particularly if you find any questions unclear.

Researcher considerations

1. Please detail the involvement that researchers who are based in the region(s) of study had during a) study design; b) clinical study processes, such as processing blood samples, prescribing medication, or patient recruitment; c) data interpretation; and d) manuscript preparation, commenting on all aspects. If they were not involved in any of these aspects, please explain why.

This question is intended for international partnerships; if all your authors are based in the area of study, this question is not applicable.

This should include a thorough description of their leadership role(s) in the study. Are local researchers named in the author list or the acknowledgements, or are they not mentioned at all (and, if not, why)? Please also describe the involvement of early career researchers based in the location of the study. Some of this information might be repeated from the Contributors section in the manuscript. Note: we adhere to ICMJE authorship criteria when deciding who should be named on a paper.

**a) Study design:**<br><br>The DoRIS and KEN SHE trial investigators are committed to fostering collaborative research, and have built on successful long-term research partnerships between key institutions in Tanzania and Kenya, and institutions in the Global North. Researchers based in Tanzania (DoRIS) and Kenya (KEN SHE) were involved in the two trials from their inception as joint/site Principal Investigators or co-Investigators – including securing of funding, study design and protocol development. These individuals are all listed as authors on the paper. 11 of the 19 authors are based in East Africa. Of the 8 joint/site Principal Investigators for the two trials, 6 are based in East Africa.
**b) Clinical study processes:**<br><br>The clinical processes for both trials were conducted and overseen by researchers in Tanzania and Kenya, including participant recruitment, questionnaire administration and clinical examinations at follow-up visits, and processing of blood samples. Although the specialist VLP ELISA assays for the

immunobridging analysis were conducted in the United States, many of the other laboratory tests were done in Tanzania or Kenya, include HPV genotyping for KEN SHE. The researchers overseeing the clinical processes are included as authors on the paper.

c) Data interpretation:

Researchers based in Tanzania and Kenya had a key role in data interpretation.

d) Manuscript preparation:

The author who prepared the first draft of the manuscript was based in East Africa for many years. All authors contributed to revisions of the manuscript and approved the final submitted version, including the 11 who are based in East Africa.

2. Were the data used in your study collected by authors named on the paper, or have they been extracted from a source such as a national survey? ie, is this a secondary analysis of data that were not collected by the authors of this paper. If the authors of this paper were not involved in data collection, how were data interpreted with sufficient contextual knowledge?

The Lancet Global Health *believe contextual understanding is crucial for informed data analysis and interpretation.*

The data were collected by authors who are named on the paper, as described in the previous section.

3. How was funding used to remunerate and enhance the skills of researchers and institutions based in the area(s) of study? And how was funding used to improve research infrastructure in the area of study?

Potentially effective investments into long-term skills and opportunities within institutions could include training or mentorship in analytical techniques and manuscript writing, opportunities to lead all or specific aspects of the study, financial remuneration rather than requiring volunteers, and other professional development and educational opportunities.

Improvements to research infrastructure could be funding of extended trial designs (such as platform trials) and use of master protocols to enable these designs, establishment of long-term contracts for research staff, building research facilities, and local control of funding allocation.

Skills:

Research capacity strengthening was built into the funding applications for both trials. DoRIS and KEN SHE study staff received training in all aspects of clinical trials, including but not limited to GCP, clinical trial management, protocol training, informed consent processes, SOP writing, reporting SAEs, sample collection and processing, quality assurance, maintenance of an

investigator site file, GCLP, immunology training, dealing with data queries, and community liaison activities. Clinical and laboratory staff have been mentored and supported to lead aspects of the trials, and to attend and present at scientific conferences. Local investigators have also completed formal training such as Masters in Public Health (MPH). The DoRIS trial has been extended to follow participants for 9 years and we have appointed one of the clinical team members to the post of trial manager for this trial extension.
**Research infrastructure:**<br><br>The Mwanza Intervention Trials Unit (MITU) in Mwanza (where the DoRIS trial was conducted) is supported by a major UK partner institution (LSHTM) as part of a successful and equitable long-term (>30 years) collaboration with the Tanzanian National Institute for Medical Research (NIMR). The MITU research facilities were built in 2008 with funding from the UK Medical Research Council (MRC), secured through this partnership. MITU is an integral component of NIMR Mwanza Centre and administers its own funding. Through the KEN SHE funding, infrastructure for clinics, generators, and -80 C freezers were established. Also, the pharmacies strengthened their infrastructure for vaccine trials, which were deployed during the COVID-19 pandemic.

4. How did you safeguard the researchers who implemented the study?

   Please describe how you guaranteed safe working conditions for study staff, including provision of appropriate personal protective equipment, protection from violence, and prevention of overworking.

Both DoRIS and KEN SHE study staff follow standard operating procedures for safe processes at work including health and safety measures in the clinics and laboratories. Oversight of procedures, including safety, was also conducted. All equipment is subject to annual servicing and checks. Staff were provided with appropriate personal protective equipment for blood draws and laboratory processes and this was expanded during the COVID-19 pandemic to include masks, etc. Training has been given on sensitive issues such as sexual harassment in the workplace. The DoRIS trial clinic site is located in a secure compound owned by the National Institute for Medical Research in Mwanza. Premises have local security staff on duty. Hours of work are logged into the register at MITU which is monitored by HR staff and the key line manager. Staff whose duties covered weekends and public holidays are given time off in lieu or paid overtime. For KEN SHE, KEMRI guidelines were closely followed at all times, including during the COVID-19 lockdowns.

Benefits to the communities and regions of study

5. How does the study address the research and policy priorities of its location?

   How were the local priorities determined and then used to inform the research question? Who decided which priorities to take forward? Which elements of the study address those priorities?

East Africa has one of the highest rates of cervical cancer incidence and mortality in the world. HPV vaccine scale-up has the potential to dramatically decrease cervical cancer cases in the long-term. However, at the time that the two trials were planned, neither Tanzania nor Kenya had a

national HPV vaccination programme, in part because of the high cost and logistical complexities of delivering a multi-dose schedule. The DoRIS trial followed previous work on HPV vaccine led by MITU which helped inform early Ministry of Health (MoH) policies on HPV vaccine introduction. The DoRIS and KEN SHE trials were designed in consultation with the MoH of both countries, as they were developing their national cervical cancer control strategies and discussing how HPV vaccination might be included. The results of both trials are critical for each country's decision-making regarding their HPV vaccine programmes. The Tanzanian and Kenyan MoH were kept informed and involved during all stages of the trials to ensure that the results that they needed to inform national policies and recommendations were available. The study results have directly informed policy in both countries (and beyond).

6. How will research products be shared in the community of study?

For instance, will you be providing written or oral layperson summaries for non-academic information sharing? Will study data be made available to institutions in the region(s) of study? The Lancet Global Health *encourages authors to translate the summary (abstract) into relevant languages after paper editing; do you intend to translate your summary?*

The investigators of both trials are fully committed to sharing and dissemination of study results. We believe that community and stakeholder consultations are important at every stage of a research study, in order to build and sustain trust in research and research partnerships.
We worked closely with community stakeholders throughout the trials. A community advisory board (CAB) was established, with local leaders, trial participants, parents/guardians, health workers, teachers and other community members invited to join. Regular community and stakeholder meetings were held during the trials, to disseminate findings and information on study progress. Extensive dissemination of the DoRIS results was done with schools and communities in 2023. The study results have been presented at local and national meetings, and the abstract of the manuscript will be translated into KiSwahili.

7. How were individuals, communities, and environments protected from harm?

a) *How did you ensure that sensitive patient data was handled safely and respectfully? Was there any potential for stigma or discrimination against participants arising from any of the procedures or outcomes of the study?*

The investigators of both trials have extensive experience in data protection and protection of privacy of participants. Study staff were specifically trained in preserving confidentiality of trial participants, including training in Good Clinical Practice and Human Subjects Protection. All interviews were conducted in private and confidential settings. Interview and clinical data, questionnaires, laboratory, and other trial forms were identified by unique study ID numbers, with no personal identifiers to maintain participant confidentiality. Personal identifiers (name, address) were only collected for informed consent, and for tracing of participants by the study team. A linking list with study ID numbers and personal identifiers was kept at the clinic, separate from the other documents, and was only accessible to selected research team members (such as the lead clinicians).

Participants were asked questions about sexual behaviour and genital hygiene practices that may have potentially resulted in embarrassment or distress. Interviewers were experienced in sexual and reproductive health research, and received specialised training in handling these sensitive data. We also tested for HIV, so there could have been psychological trauma from learning one's HIV status. Nurses, counsellors and other relevant staff had specialised training in counselling about HIV, STIs, cervical cancer screening and treatment, stigma, and gender-based issues.

Staff who conducted the informed consent process were fully trained with specific training around discussion of key messages and questions to ensure that potential participants understood the study before agreeing to participate.

Although every effort to protect participant privacy and confidentiality was made, it was theoretically possible that social harms could have resulted owing to an individual's participation in the study (e.g. through accidental disclosure of a participant's HIV status). Both trials had plans for assessing study-related social harms and referring participants to appropriate resources as needed.

b) *Might any of the tests be experienced as invasive or culturally insensitive?*

The collection of genital swabs for HPV testing may have been considered invasive or culturally insensitive, particularly for young girls (the DoRIS trial). In the DoRIS trial (age 9-14 years), self-administered swabs were collected, with assistance of an experienced nurse, to minimise discomfort and embarrassment. This method was developed and evaluated carefully through focus group discussions with adolescent girls. We have used this method in many of our previous studies of HPV and sexual and reproductive health in this age group, and results have been published showing high acceptability of this approach. The procedure was carefully explained with verbal, diagrammatic and written information in Swahili. In the KEN SHE trial (age 15-25 years, all sexually active), swabs were collected during a pelvic examination by an experienced clinician; procedures were explained, questions answered, and the participants were given a choice to have a chaperone present for the examination.

c) *How did you determine that work was sensitive to traditions, restrictions, and considerations of all cultural and religious groups in the study population?*

Input from key stakeholders was sought to help guide the development of the study protocol, standard operating procedures, and plans for sensitisation. Before the studies began, we held focus group discussions with community members (parents, health workers, religious leaders, teachers, sexually active young women and non-sexually active women) to explore community attitudes towards HPV vaccination and other study procedures and during the study we conducted qualitative research with parents and participants that helped inform our ongoing engagement with participants.

d) *Were biowaste and radioactive waste disposed of in accordance with local laws?*

Yes

e) *Were any structures built that would have impacted members of the community or the environment (such as handwashing facilities in a public space)? If so, how did you ensure that you had appropriate community buy-in?*

Not applicable

f) *How might the study have impacted existing health-care resources (such as staff workloads, use of equipment that is typically employed elsewhere, or reallocation of public funds)?*

The DoRIS and KEN SHE trials did not carry out data collection in existing health care facilities. Staff were specifically employed to work on the study and the study used equipment that was in place in MITU/KEMRI or purchased specifically for the study. No public funds were reallocated for the either trial.

8. Finally, please provide the title (eg, Dr/Prof, Mr/Mrs/Ms/Mx), name, and email address of an author who can be contacted about this statement. This can be the corresponding author.

**Name:**	Kathy Baisley
**Email:**	kathy.baisley@lshtm.ac.uk
